# Predicting global horizontal irradiance using regression-based machine learning models in high-altitude environments

**DOI:** 10.3389/frai.2026.1870055

**Published:** 2026-09-14

**Authors:** Milton Edward Humpiri-Flores, David Mamani-Pari, Danny Lévano, Roel Dante Gomez Apaza, Esteban Tocto-Cano, Ángel Rosendo Condori-Coaquira, Javier Linkolk López-Gonzales

**Affiliations:** 1 Escuela Profesional de Ingeniería de Sistemas, Universidad Peruana Unión, Juliaca, Peru; 2 Escuela de Posgrado, Universidad Peruana Unión, Lima, Peru

**Keywords:** global horizontal irradiation, high-altitude Andes, neural networks, solar radiation forecasting, target leakage

## Abstract

Global solar radiation is a fundamental component of the Earth's energy balance and plays a critical role in integrating renewable energy into electrical systems. However, its pronounced variability, particularly in high-altitude regions, constrains energy stability and complicates planning processes. This study evaluated regression-based and machine-learning models for 1-day-ahead prediction of global horizontal irradiance (GHI) in Juliaca, Peru (>3,800 m.a.s.l.), using 11 years (2010–2020) of daily NASA POWER data (*n* = 4,000 observations after cleaning; 80/20 chronological train/test split: training from 2010 to 2018 and testing from 2018 to 2020). During data auditing, we identified that the same-day clearness index (*K*_*T*_) is algebraically related to the same-day GHI through the extraterrestrial radiation term, which would otherwise inflate the apparent predictive accuracy. Therefore, we restricted same-day predictors to cyclical solar geometry terms and used lagged specific humidity, clearness index, and irradiance values from the previous 1–3 days as legitimate autoregressive information. Under this leakage-free design, Linear Regression achieved the best performance (*R*^2^ = 0.390, MAE = 0.677 kWh m^−2^d^−1^, and RMSE = 0.896 kWh m^−2^d^−1^), narrowly outperforming gradient boosting (*R*^2^ = 0.384) and polynomial regression (*R*^2^ = 0.381). Random Forest and shallow neural networks followed closely, with all six models exhibiting remarkably comparable performance. Feature-importance and SHAP analyses for the Random Forest model showed that previous-day irradiance and previous-day (lag-1) humidity were the dominant predictors. This pattern may be consistent with short-persistence cloud processes; however, cloud observations are required to verify this interpretation directly. The modest but realistic explanatory power obtained here, together with the diagnostic procedure used to achieve it, offers a template for detecting and avoiding under-reported leakage risk in solar radiation forecasting studies that use clearness-index-derived predictors.

## 1 Introduction

The transition to sustainable energy systems is a global priority because of the environmental impacts of fossil fuel consumption, particularly climate change and its effects on ecosystems and human health (Pachauri and Meyer, 2014). Non-conventional renewable sources, including solar, wind, and geothermal energy, represent economically competitive alternatives, supported by technological advances that facilitate their integration into electrical grids (Infield and Freris, 2020). This transition addresses the reduction of greenhouse gas emissions while supporting economic sustainability and political stability, with solar and wind energy constituting the central pillars of energy sector decarbonization (International Energy Agency, 2020). Despite these advances, structural inefficiencies persist, with 30%–40% of planned energy projects remaining unimplemented owing to economic or resource constraints (Tampubolon et al., 2026). These inefficiencies generate political and economic pressures and compromise grid stability, leading to supply interruptions, reduced quality, and energy losses. The integration of renewable sources into the energy mix introduces technical challenges owing to their intermittent and variable nature, making accurate solar radiation prediction essential for optimizing smart grid operations and ensuring supply reliability (Tsai et al., 2023). Solar energy is a strategic resource owing to its capacity to meet the increasing global demand for electricity. However, their generation depends on uncertain factors, including meteorological conditions and geographic characteristics, which introduce substantial variability in the data. Traditional estimation methods based on empirical or statistical models exhibit limitations in representing complex atmospheric dynamics. Machine learning approaches improve predictive performance by integrating multiple meteorological variables and capturing non-linear relationships (Teke et al., 2015; Yang et al., 2023). This study evaluated regression-based approaches to estimate solar radiation, given their relevance for accurate quantitative predictions in energy planning. Artificial neural networks are particularly effective because of their capacity to model complex non-linear relationships (Pang et al., 2020). Similarly, Random Forest algorithms provide robust performance without strict distributional assumptions, making them suitable for modeling solar radiation variability (Breiman, 2001). Machine learning and artificial intelligence are effective tools for addressing challenges in renewable energy prediction. These methods process large datasets and identify complex patterns in systems with high variabilities. Empirical studies have shown that models such as Random Forest, Gradient Boosting, and deep neural networks improve solar radiation forecasting by capturing non-linear interactions among meteorological variables. Recent studies have reported substantial gains in solar-irradiance forecasting accuracy using ensemble and deep learning approaches (Gupta et al., 2024; Nastic et al., 2024; Zhang et al., 2025; Weyll et al., 2024; Nadeem et al., 2024). These advances enhance the resilience of smart grids by improving their capacity to anticipate and respond to energy variability, particularly in hybrid systems (Weyll et al., 2024). They also inform public policy design for clean energy integration in regions with high climatic variability (Tsai et al., 2023). Most studies have focused on low- and mid-altitude contexts, leaving a gap in our understanding of model performance under high-altitude conditions, where low air density, high ultraviolet irradiance, and convective cloud dynamics alter atmospheric processes (Quispe Huamán and Sotomayor Alzamora, 2022; Demir, 2025). This gap is critical in regions such as the Peruvian Andes, where high solar potential coincides with limited predictive characterization under extreme conditions. In this study, six regression and machine-learning models were developed and evaluated for 1-day-ahead prediction of daily global horizontal irradiation at a high-altitude Andean site in Juliaca, Peru (>3,800 m a.s.l.). The leakage-controlled predictor set comprised specific humidity lagged by 1–2 days, irradiance values lagged by 1–3 days, clearness-index values lagged by 1–2 days, and cyclical solar geometry terms. This study also examined the effect of excluding same-day target-derived predictors that may artificially inflate the forecasting performance. Accurate prediction enhances smart grid efficiency and supports stable energy management, positioning renewable energy forecasting as a critical component of resilient and sustainable power systems. Accordingly, this study addresses the following research question: To what extent can six regression and machine-learning models predict next-day daily global horizontal irradiation at Juliaca using only predictors considered available at forecast issuance, and which model provides the best out-of sample performance under this leakage-controlled design? Several recent studies have compared Random Forest with other regression or deep-learning models for solar-irradiance forecasting, including in high altitude or high-elevation settings (Gupta et al., 2024; Nastic et al., 2024; Zhang et al., 2025; Weyll et al., 2024; Nadeem et al., 2024). Therefore, applying these algorithms to a new location is not the principal contribution of the present study. Instead, the novel contribution is methodological: this study shows that a same-day clearness-index predictor is algebraically related to the target irradiance through the extraterrestrial radiation term, quantifies the resulting inflation of apparent accuracy (*R*^2^ = 0.95 from a two-variable model containing only same-day *K*_*T*_ and day of year), and proposes a leakage controlled predictor-selection procedure that can be applied to other clearness-index-based forecasting studies, regardless of location or altitude. The remainder of this paper is structured as follows: Section 2 describes the methodology, Section 3 presents the results and discussion, and Section 4 concludes the study.

## 2 Methodology

Advances in satellite technology have enhanced the accuracy of monitoring climatic variables that determine solar radiation dynamics. Satellite platforms generate high-resolution datasets that enable the characterization of key parameters, including solar geometry, Earth-Sun distance, and atmospheric conditions that influence the photovoltaic conversion efficiency. Within this framework, the application of machine learning (ML) techniques improves the prediction of available solar radiation, thereby supporting the optimization of renewable energy generation (Teke et al., 2015). Accordingly, the methodology was structured into sequential phases designed to maximize the predictive performance of the proposed models (Figure 1).

**Figure 1:**
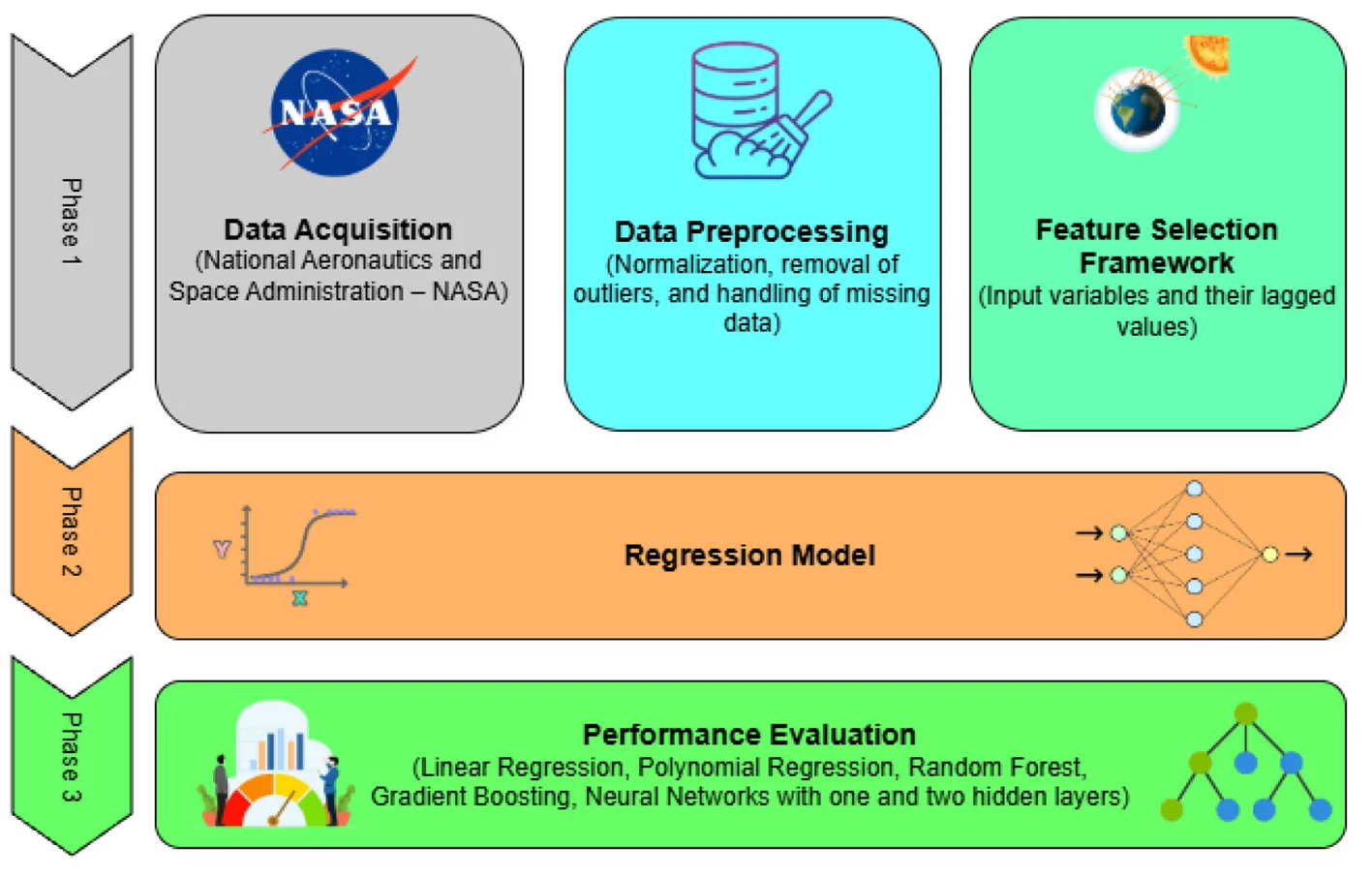
Methodological framework for solar radiation prediction. Flowchart illustrating the three main phases of the study: (Phase 1) Data acquisition from NASA, preprocessing (normalization and outlier handling), and feature selection using lag values; (Phase 2) Implementation of prediction models; (Phase 3) Model evaluation and selection based on performance metrics for six regression and machine-learning models, including linear and polynomial regression, Random Forest, Gradient Boosting, and feedforward neural networks with one and two hidden layers.

The forecasting framework comprises five sequential stages (Figure 1). First, the daily solar radiation and meteorological data were obtained from NASA POWER. Second, the data were audited, cleaned, chronologically ordered, and transformed to construct the lagged predictors. Third, the leakage audit excluded DTI and same-day clearness index from the final predictor set, while retaining lagged irradiance, lagged clearness index, lagged specific humidity, and cyclical day-of-year terms. Fourth, the data were divided chronologically into an 80% training set and a 20% held-out test set. The hyperparameters were selected using a three-fold TimeSeriesSplit cross-validation applied exclusively to the training set. Finally,

six models were evaluated during the held-out test period using MAE, RMSE, MAPE, SMAPE, and *R*^2^.

Peru exhibits high levels of solar radiation owing to its tropical latitude, resulting in consistently high solar incidence throughout the year. This national-scale characteristic is distinct from the altitude-specific mechanisms examined in this study. As an illustration of Peru's solar potential at the national level, the ultraviolet index values reached 15 in Lima, a coastal city located near sea level, during the summer of 2016 (AutoSolar, 2025). This example is included solely to demonstrate the overall solar potential of the country and should not be interpreted as evidence of the Andean high-altitude dynamics. These dynamics are associated with reduced atmospheric density and constitute the specific focus of the Juliaca analysis presented in this study.

### 2.1 Data acquisition

This study employed regression models and machine learning algorithms to predict solar radiation using datasets from NASA's Global Energy Resource Prediction Program (NASA, 2025) (Table 1). The dataset includes key meteorological variables, such as the downward thermal infrared radiation flux, specific humidity, and clearness index, which serve as predictors of solar radiation. The response variable is defined as the global solar radiation (insolation) at the Earth's surface (Table 2), providing a consistent analytical basis for evaluating the predictive model performance.

**Table 1:** Visualization of the meteorological and solar radiation dataset for Juliaca, Peru (2010–2020).

Index	LAT	LON	YEAR	MO	DY	QV2M	KT	DTI	SII
1	−15.488	−70.149	2010	1	1	0.0696	0.70	148.76	8.02
2	−15.488	−70.149	2010	1	2	0.0812	0.70	148.48	7.94
3	−15.488	−70.149	2010	1	3	0.0879	0.61	130.47	6.96
4	−15.488	−70.149	2010	1	4	0.0885	0.62	131.64	7.01
5	−15.488	−70.149	2010	1	5	0.0818	0.64	136.66	7.32
6	−15.488	−70.149	2010	1	6	0.0787	0.54	116.93	6.17
7	−15.488	−70.149	2010	1	7	0.0800	0.71	150.55	8.08
8	−15.488	−70.149	2010	1	8	0.0812	0.61	129.30	6.91
…									
4017	−15.488	−70.149	2020	12	30	0.0879	0.41	90.21	4.68
4018	−15.488	−70.149	2020	12	31	0.0836	0.36	79.55	4.07

**Table 2:** Available dataset for solar radiation forecasting.

Variable	Type	Description	Abbreviation	Unit
Latitude	Float	Geographic latitude coordinate in decimal degrees	LAT	°
Longitude	Float	Geographic longitude coordinate in decimal degrees	LON	°
Year	Integer	Year of the recorded measurement	YEAR	–
Month	Integer	Month of the recorded measurement	MO	–
Day	Integer	Day of the recorded measurement	DY	–
Relative humidity	Float	Relative humidity at 2 m above ground level	QV2M	Fraction
Clearness index	Float	Sky clearness index	KT	–
Dew point temperature index	Float	Dew point temperature index	DTI	°C
Solar irradiance	Float	Solar irradiance in watts per square meter	SII	W/m^−2^

#### 2.1.1 Key factors contributing to high solar radiation in Peru

Peru exhibits high levels of solar radiation owing to its tropical location, resulting in consistently high solar incidence throughout the year. This effect intensifies in the Andean high-altitude regions, where reduced atmospheric density limits absorption and scattering processes, thereby increasing the amount of irradiance that reach the surface. However, the magnitude of solar radiation is modulated by local factors, including cloud cover and aerosol concentration, which introduce spatial and temporal variability even in coastal areas.

As shown in Figure 2, the average monthly solar radiation recorded during the 2010–2020 period exhibits a clear seasonal pattern. Higher irradiance values occurred mainly toward the end and beginning of the year, whereas lower values were observed during the middle of the year. This variation reflects the combined influence of seasonal solar geometry and local atmospheric conditions. Empirical evidence further supports Peru's elevated radiation context; for instance, ultraviolet index values reached 15 in Lima during the summer of 2016, accompanied by maximum air temperatures near 37 °C and heat index values of up to 42 °C during peak insolation periods (AutoSolar, 2025). Collectively, these factors demonstrate Peru's substantial solar energy potential, although the variability illustrated in Figure 2 highlights the need for detailed temporal and spatial characterizations to support efficient energy planning and utilization.

**Figure 2:**
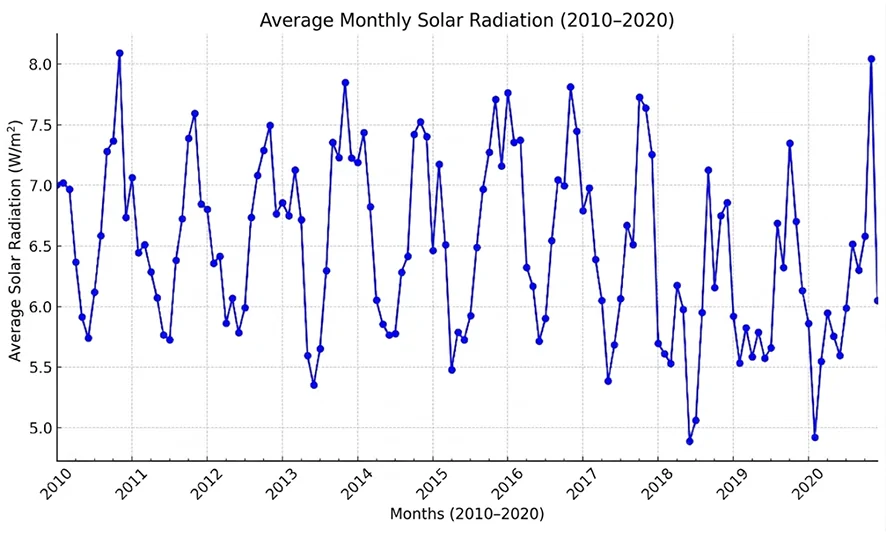
Average monthly solar radiation (2010–2020). Monthly mean solar radiation calculated from observations recorded between 2010 and 2020. The line with circular markers illustrates the seasonal variation in surface irradiance throughout the year.

### 2.2 Data preprocessing

The dataset comprised 4,018 daily records collected between January 1, 2010 and December 31, 2020. After removing 15 duplicate calendar-date entries, 4,003 unique daily observations remained in the dataset for analysis. The construction of predictors lagged by 1–3 days reduced the analysis-ready sample to 4,000 observations. All records were daily aggregates, and no sub-daily observations were used in this study.

The predictor variables represent the key atmospheric processes that govern the surface solar radiation. The downward thermal infrared radiative flux quantifies atmospheric emissions resulting from absorption, emission, and scattering processes, expressed in kilowatt-hours per square meter per day (kWh m^−2^ d^−1^) within the 4–100 μm spectral range. The specific humidity at 2 m measures water vapor content (g kg^−1^) and exhibits a negative association with solar radiation owing to its absorptive properties, which can reduce photovoltaic efficiency (Panjwani and Narejo, 2014). The clearness index (*K*_*t*_) represents atmospheric transmissivity as the ratio of surface to extraterrestrial radiation, expressed as a dimensionless value between 0 and 1 (Wald, 2018). Collectively, these variables provide a physically consistent basis for modeling solar radiation variability. Photovoltaic energy generation primarily depends on incident solar radiation, which determines the irradiance received by the panel. The energy output is directly proportional to this irradiance for a given system configuration, making solar radiation a critical variable in yield estimation. Accordingly, the photovoltaic output can be represented by a functional relationship that integrates the incident radiation, system characteristics, and conversion efficiency (Photovoltaic Software, 2025).


E=A·r·H·PR

The variables used in the photovoltaic energy estimation model are defined as follows: *E* denotes the energy generated (kWh), *A* represents the total surface area of the solar panel (m^2^), and *r* indicates the panel efficiency (%). The variable *H* corresponds to the average annual solar radiation incident on tilted panels, excluding shading effects, while *PR* denotes the performance ratio, which accounts for system losses and typically ranges between 0.5 and 0.9, with a standard value of 0.75.

### 2.3 Feature selection framework

Table 3 summarizes the distribution and scale of the four principal variables considered during the initial feature selection stage. The target variable, solar irradiance (SII), had a mean value of 6.49 and a standard deviation of 1.07, whereas the clearness index (*K*_*T*_) had a mean of 0.68 and a standard deviation of 0.11. The downward thermal infrared flux (DTI) exhibited substantially greater numerical dispersion, with values ranging from 15.77 to 167.17. These descriptive statistics provide an empirical context for evaluating the relationships among the candidate predictors and identifying the variables that may contain redundant or target-derived information.

**Table 3:** Descriptive statistical characteristics of the dataset variables

Variable	Mean	Std. Dev.	Min	Max	25%	50%	75%
QV2M	0.06	0.01	0.01	0.09	0.05	0.06	0.07
KT	0.68	0.11	0.10	0.86	0.61	0.70	0.78
DTI	118.30	19.26	15.77	167.17	104.80	116.90	131.90
SII	6.49	1.07	0.77	9.35	5.82	6.42	7.21

The Pearson correlation matrix presented in Figure 3 reveals a particularly strong association between DTI and SII. The corresponding Pearson coefficient was *r* = 0.989, indicating that the two variables exhibited almost identical linear variations within the analyzed dataset. Because DTI and SII are measured concurrently and are not connected through a known deterministic physical identity, such an unusually high correlation between nominally distinct atmospheric fluxes suggests a substantial information redundancy. Including both variables in the predictive models could therefore inflate the apparent model performance and obscure the independent effects of the remaining atmospheric variables.

**Figure 3:**
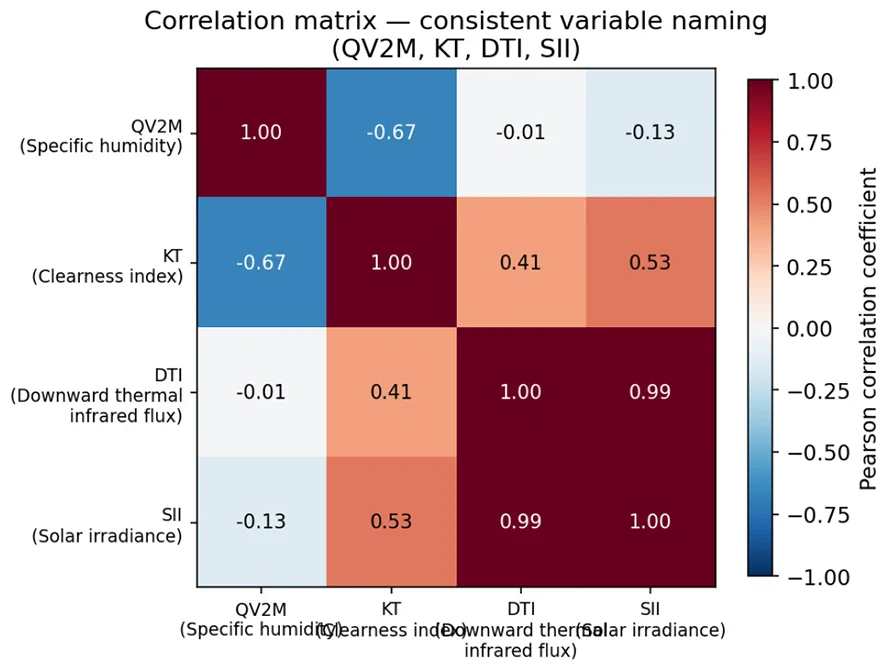
Pearson correlation matrix of the principal dataset variables. The matrix shows the linear associations among specific humidity (QV2M), clearness index (*K*_*T*_), downward thermal infrared flux (DTI), and daily solar irradiance (SII). The near-perfect DTI–SII correlation indicates substantial redundancy between these concurrently measured variables.

To determine whether this association was specific to linear correlation or reflected a broader monotonic relationship, Pearson and Spearman coefficients were compared Figure 4. The DTI–SII relationship remained virtually unchanged under both measures, with *r* = 0.989 for Pearson's coefficient and *r*_*s*_ = 0.986 for Spearman's coefficient. Their close agreement indicates that the observed dependence is not an artifact of a non-linear relationship captured by only one correlation measurement.

**Figure 4:**
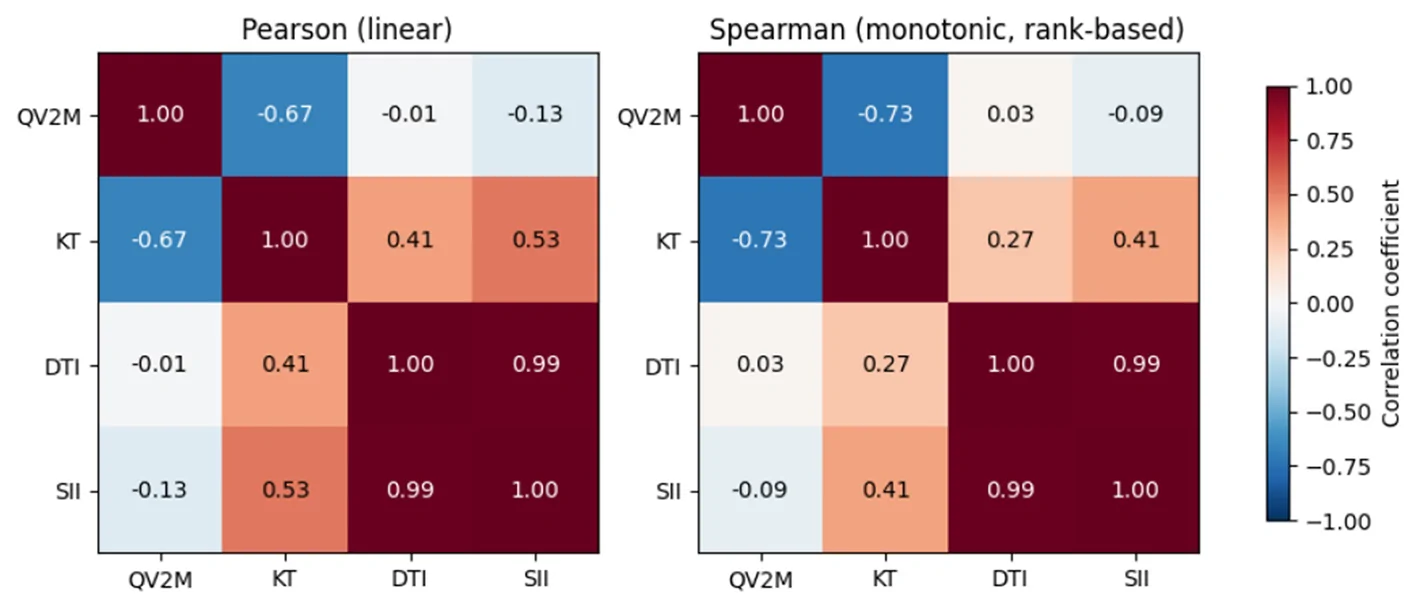
Comparison of Pearson and Spearman correlation coefficients. The close agreement between the linear and rank-based coefficients, particularly for DTI and SII (*r* = 0.989 and *r*_*s*_ = 0.986, respectively), confirms that their strong association is robust.

A more consequential feature-selection issue concerns the clearness index, defined as


$$K_{T}=\frac{\text{SII}}{H_{0}},$$

where *H*_0_ denotes extraterrestrial radiation, which is a deterministic function of latitude and day of the year. Thus, same-day *K*_*T*_ directly includes the target variable SII in its definition. When same-day *K*_*T*_ is combined with calendar or solar-geometry variables that approximate or determine *H*_0_, predicting the same-day SII becomes equivalent to approximately reconstructing a known algebraic identity rather than learning an empirical relationship between atmospheric conditions and solar radiation.

This leakage risk is consistent with the strong association between *K*_*T*_ and SII Figure 3. To verify its practical effect, an ordinary least squares model was fitted using only same-day *K*_*T*_ and day of year. The model obtained *R*^2^ = 0.95, which is implausibly high for a genuine 1-day-ahead solar-radiation forecast in a high-altitude environment characterized by substantial atmospheric variability. This result confirms that the apparent predictive performance primarily arises from target-derived information rather than from legitimate forecasting capacity.

This is based on the statistical evidence summarized in Table 3 and the correlation patterns documented in Figures 3, 4, DTI was excluded because of its extreme redundancy with SII. Same-day *K*_*T*_ was excluded because it is algebraically derived from the target variable. Only lagged clearness-index values, *K*_*T, t*−1_ and *K*_*T, t*−2_, were retained, as they represent historical information available before the forecast day and therefore do not introduce direct target leakage. Specific humidity, lagged by 1 and 2 days (QV2M_*t*−1_ and QV2M_*t*−2_), was retained as an independently measured atmospheric variable that was not mathematically derived from the SII. The same-day specific humidity was not included in the final predictor set; to keep the leakage-control design conservative and internally consistent, every meteorological predictor other than the cyclical solar geometry terms was restricted to lagged (pre-forecast-day) values, even though no algebraic leakage mechanism analogous to that of *K*_*T*_ was identified for humidity.

### 2.4 Models used

Solar radiation forecasting relies on statistical and machine learning models with different capacities to represent linear, non-linear, and temporal relationships. Linear regression provides a baseline approach owin to its structural simplicity and interpretability; however, it remains limited to linear or near-linear dynamics, restricting its applicability in complex atmospheric systems (Menacho-Chiok, 2014; Gala et al., 2016; Wei, 2017). Polynomial regression extends this framework by incorporating higher-order terms to capture non-linear patterns, although this flexibility increases the risk of overfitting and reduces generalizability (Bamisile et al., 2022; Quispe Huamán and Sotomayor Alzamora, 2022). Time series models such as autoregressive integrated moving average (ARIMA), represent temporal dependencies through autoregressive, integration, and moving average components; however, their reliance on linearity and stationarity assumptions constrains their performance under highly variable conditions (Chodakowska et al., 2023; Belmahdi et al., 2023; Alsharif et al., 2019). In contrast, artificial neural networks model complex non-linear interactions and stochastic relationships, albeit with high data requirements and susceptibility to overfitting (He et al., 2016). The multilayer perceptron (MLP), as a universal function approximator, captures non-linear transformations through layered structures, although it entails significant computational costs and is sensitive to data quality (Rumelhart et al., 1986). Architectures with one or two hidden layers reduce model complexity and overfitting risk but limit representational depth compared to deeper networks (Hinton et al., 2006). Ensemble methods further enhance predictive capacity: Random Forest reduces variance through the aggregation of multiple decision trees without imposing parametric assumptions, whereas Gradient Boosting iteratively minimizes prediction error through sequential learning, albeit with sensitivity to hyperparameter tuning (Weyll et al., 2024; Friedman, 2001). Collectively, these approaches illustrate the trade-off between interpretability, flexibility, and predictive performance in solar radiation modeling.

Two feedforward neural-network architectures were evaluated: one model with a single hidden layer and another with two hidden layers. All six models were tuned using a grid search with a three-fold TimesSeriesSplit cross-validation applied exclusively to the training set. The held-out test set, corresponding to the final 20% of observations in chronological order, was not used for model selection. The hyperparameter settings for all evaluated models are summarized in Table 4.

**Table 4:** Complete hyperparameter settings for all evaluated models.

Model	Key hyperparameters selected by cross-validation
Linear regression	Ridge regularization with α = 0.0001, approximately equivalent to ordinary least squares; standardized input variables
Polynomial regression	Second-degree polynomial expansion; Ridge regularization with α = 1.0; standardized input variables
Random Forest	n_estimators = 200; max_depth = None; min_samples_leaf = 5; random_state = 42
Gradient boosting	n_estimators = 100; max_depth = 2; learning_rate = 0.1; random_state = 42
Neural network (one hidden layer)	32 ReLU units; Adam optimizer; learning_rate_init = 10^−3^; *L*_2_ regularization with α = 10^−3^; early stopping with patience = 25; standardized input variables
Neural network (two hidden layers)	64 and 32 ReLU units in the first and second hidden layers, respectively; Adam optimizer; learning_rate_init = 10^−3^; *L*_2_ regularization with α = 10^−3^; early stopping with patience = 25; standardized input variables

### 2.5 Model evaluation and selection

Model evaluation employs complementary statistical metrics that capture multiple dimensions of the predictive error. The mean absolute error (MAE) measures the average magnitude of the absolute deviations between the observed and predicted values, providing a direct indicator of accuracy. The mean absolute percentage error (MAPE) expresses the prediction error in relative terms, enabling comparisons across different scales, although it becomes unstable when the observed values approach zero. The symmetric mean absolute percentage error (SMAPE) addresses this limitation by normalizing the relative error, yielding a more balanced assessment of prediction accuracy. Additionally, the coefficient of determination (*R*^2^) quantifies the proportion of variance explained by the model, with values approaching 1 indicating strong explanatory power. Together, these metrics provide a robust, multidimensional framework for evaluating and comparing the performance of predictive models.

## 3 Results and discussion

Figure 5 shows the Pearson correlation matrix for specific humidity (QV2M), clearness index (*K*_*T*_), downward thermal infrared flux (DTI), and daily global horizontal irradiation (GHI). The matrix reveals several relationships relevant to feature selection and predictive modeling. QV2M exhibited a moderate negative correlation with *K*_*T*_ (*r* = −0.67), suggesting that higher atmospheric humidity is associated with lower atmospheric transmissivity. In contrast, the correlation between QV2M and DTI was negligible (*r* = −0.01), whereas QV2M showed only a weak negative correlation with the SII (*r* = −0.13). These results indicate that humidity has a limited direct linear association with surface solar irradiation in the analyzed dataset.

**Figure 5:**
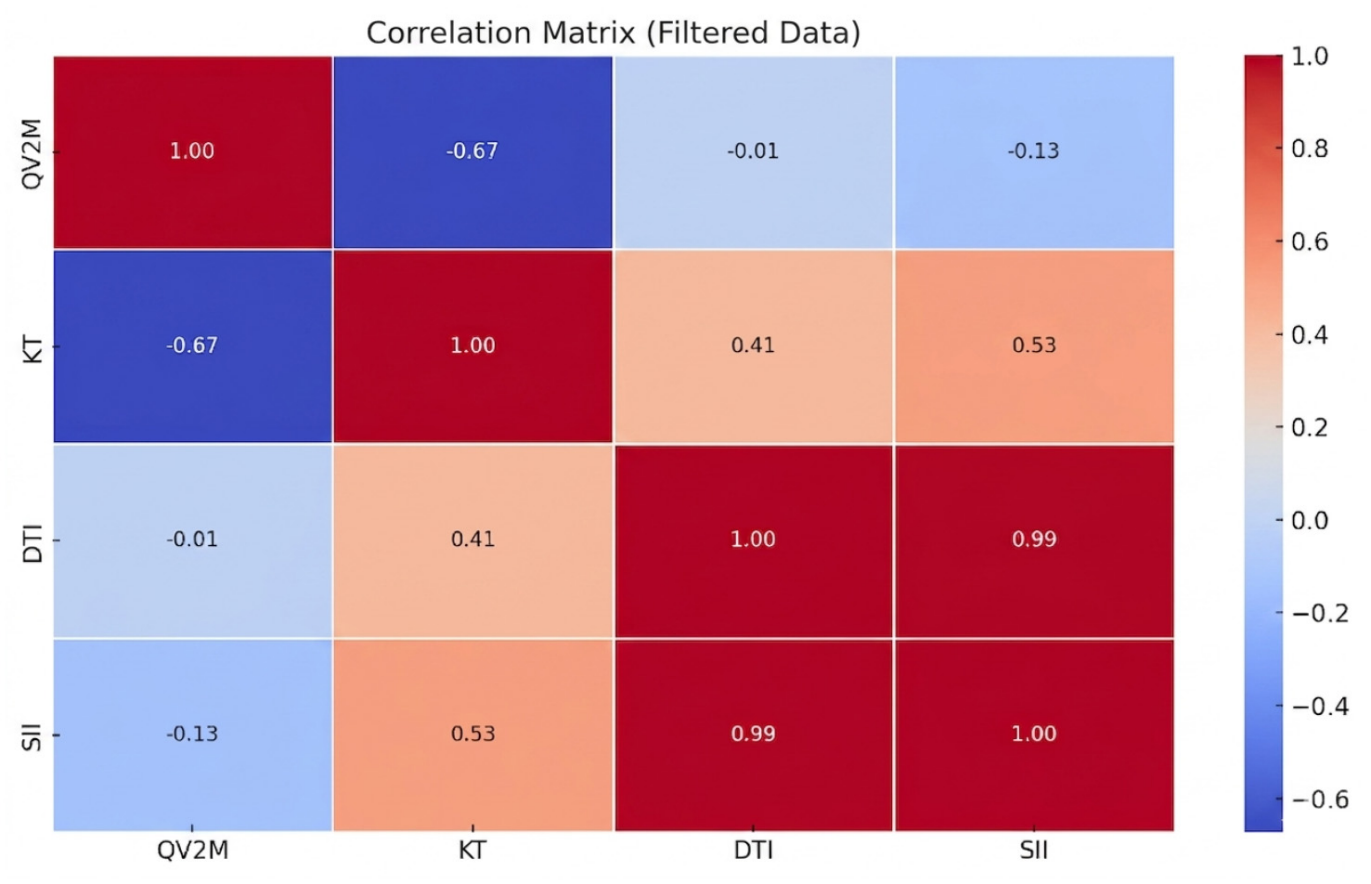
Correlation matrix. Pearson correlation coefficients among specific humidity (QV2M), clearness index (*K*_*T*_), downward thermal infrared flux (DTI), and daily global horizontal irradiation (SII). Positive correlations are represented in red and negative correlations in blue.

As shown in Figure 5, *K*_*T*_ presented moderate positive correlations with DTI (*r* = 0.41) and SII (*r* = 0.53). The strongest relationship was observed between DTI and SII, with a correlation coefficient of approximately *r* = 0.99. This near-perfect association indicates a substantial informational overlap between the two variables and suggests that their simultaneous inclusion could introduce redundancy and multicollinearity. Consequently, this correlation structure was considered during the leakage-control and feature-selection stages of the modeling process.

The predictive results summarized in Table 5 and visualized in Figure 6 were obtained using a leakage-controlled feature set. The predictors comprised specific humidity lagged by 1 and 2 days (QV2M_lag1_ and QV2M_lag2_), solar irradiation lagged by 1–3 days (SII_lag1_, SII_lag2_, and SII_lag3_); clearness index lagged by 1 and 2 days (KT_lag1_ and KT_lag2_); and the cyclical solar-geometry terms sin(day-of-year) and cos(day-of-year).

**Table 5:** Performance comparison of the six models under the leakage-controlled 1-day-ahead forecasting design.

Model	MAE	RMSE	MAPE (%)	SMAPE (%)	*R* ^2^
Linear regression	0.677	0.896	12.23	11.28	0.390
Polynomial regression	0.680	0.902	12.37	11.35	0.381
Random Forest	0.678	0.903	12.30	11.29	0.381
Gradient boosting	0.678	0.901	12.26	11.28	0.384
Neural network (one hidden layer)	0.678	0.903	12.23	11.28	0.381
Neural network (two hidden layers)	0.683	0.909	12.30	11.40	0.372

The same results are represented graphically in Figure 6.

**Figure 6:**
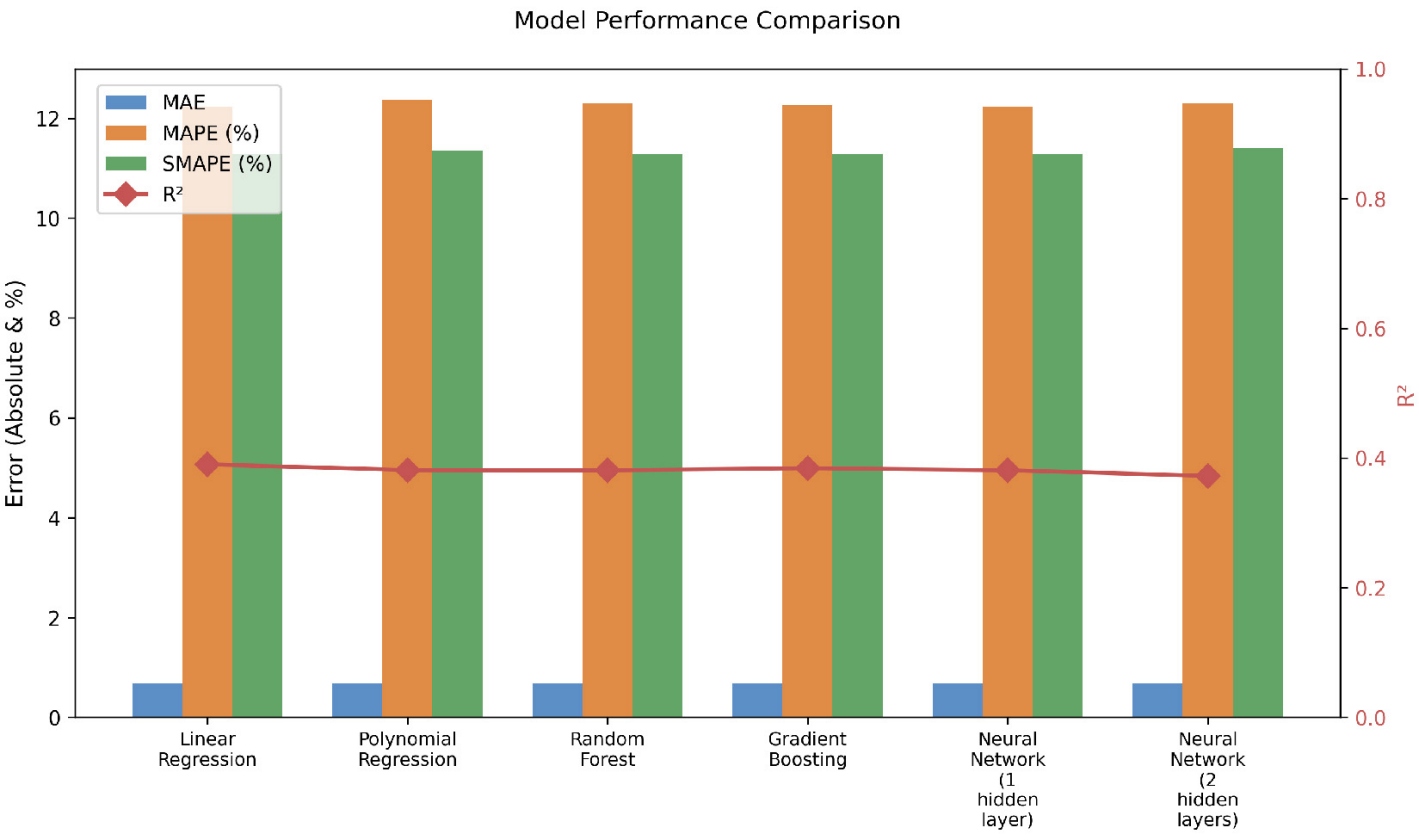
Model performance comparison. Bars represent MAE, MAPE, and SMAPE on the left axis, while diamond markers represent *R*^2^ on the right axis. The values correspond to those reported in Table 5.

A chronological 80/20 split was applied to preserve the temporal sequence of observations. The training set comprised *n* = 3, 200 daily observations recorded between 2010-01-04 and 2018-10-14, whereas the held-out test set comprised *n* = 800 observations recorded between 2018-10-15 and 2020-12-31. All performance measures reported in Table 5 and Figure 6 correspond to the same testing period.

Table 5 shows that Linear Regression achieved the best overall numerical performance, with an MAE of 0.677, an RMSE of 0.896, a MAPE of 12.23%, a SMAPE of 11.28%, and an *R*^2^ of 0.390. Gradient Boosting ranked second according to *R*^2^, reaching 0.384, with an MAE of 0.678 and an RMSE of 0.901. Random Forest and the neural network with one hidden layer also produced similar results, both obtaining an *R*^2^ of 0.381. The graphical comparison in Figure 6 reinforces the results in Table 5. The bars show that the error metrics varied only slightly across the six algorithms, while the *R*^2^ markers remained within a narrow interval from 0.372 to 0.390. Therefore, the results do not indicate the decisive superiority of any complex non-linear model.

The narrow performance range visible in Figure 6 suggests that increasing the algorithmic complexity did not produce meaningful predictive improvements in the leakage-controlled feature set. In particular, Linear Regression obtained the lowest MAE and RMSE and the highest *R*^2^, followed closely by Gradient Boosting and Random Forest. This finding indicates that once artificially inflated predictor–target relationships are removed, a comparatively simple linear model can remain competitive with ensemble and neural network approaches.

The percentage-based metrics reported in Table 5 follow a similar general pattern. Linear Regression and the neural network with one hidden layer obtained the lowest MAPE of 12.23%. Linear Regression, Gradient Boosting, and the one-hidden-layer neural network shared the lowest SMAPE of 11.28%. Nevertheless, the absolute differences were small: the MAPE ranged from 12.23 to 12.37%, whereas the SMAPE ranged from 11.28 to 11.40%. Thus, all six models produced broadly similar percentage errors during the held-out test period (Zhang et al., 2025; Nadeem et al., 2024).

Although Linear Regression achieved the best predictive performance, Random Forest was selected for the feature-importance and SHAP analyses presented in Figures 7, 8. This choice was made because the Random Forest supports non-linear, tree-based explanatory methods and achieves a test performance close to that of Linear Regression. Accordingly, these interpretability results describe the predictor structure learned by the Random Forest and should not be interpreted as explanations of the Linear Regression model.

**Figure 7:**
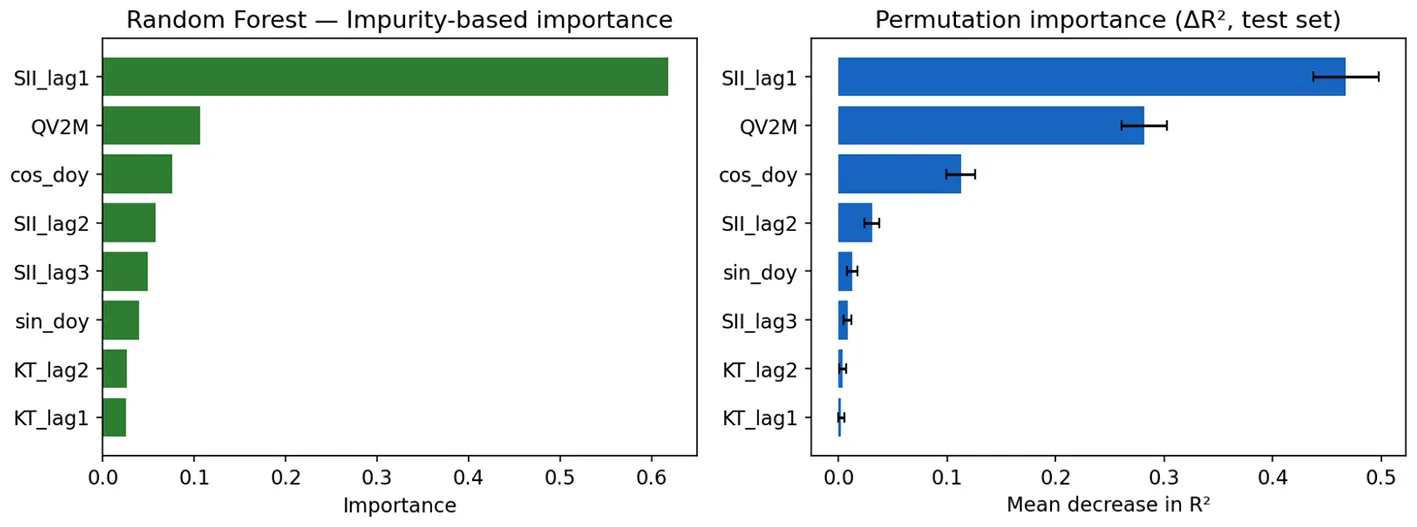
Random Forest feature importance. Impurity-based importance is presented on the left, and permutation importance calculated on the held-out test set with 30 repetitions is presented on the right. Error bars represent one standard deviation. Previous-day irradiation (SII_lag1_) and previous-day humidity (QV2M_lag1_) are the dominant predictors.

**Figure 8:**
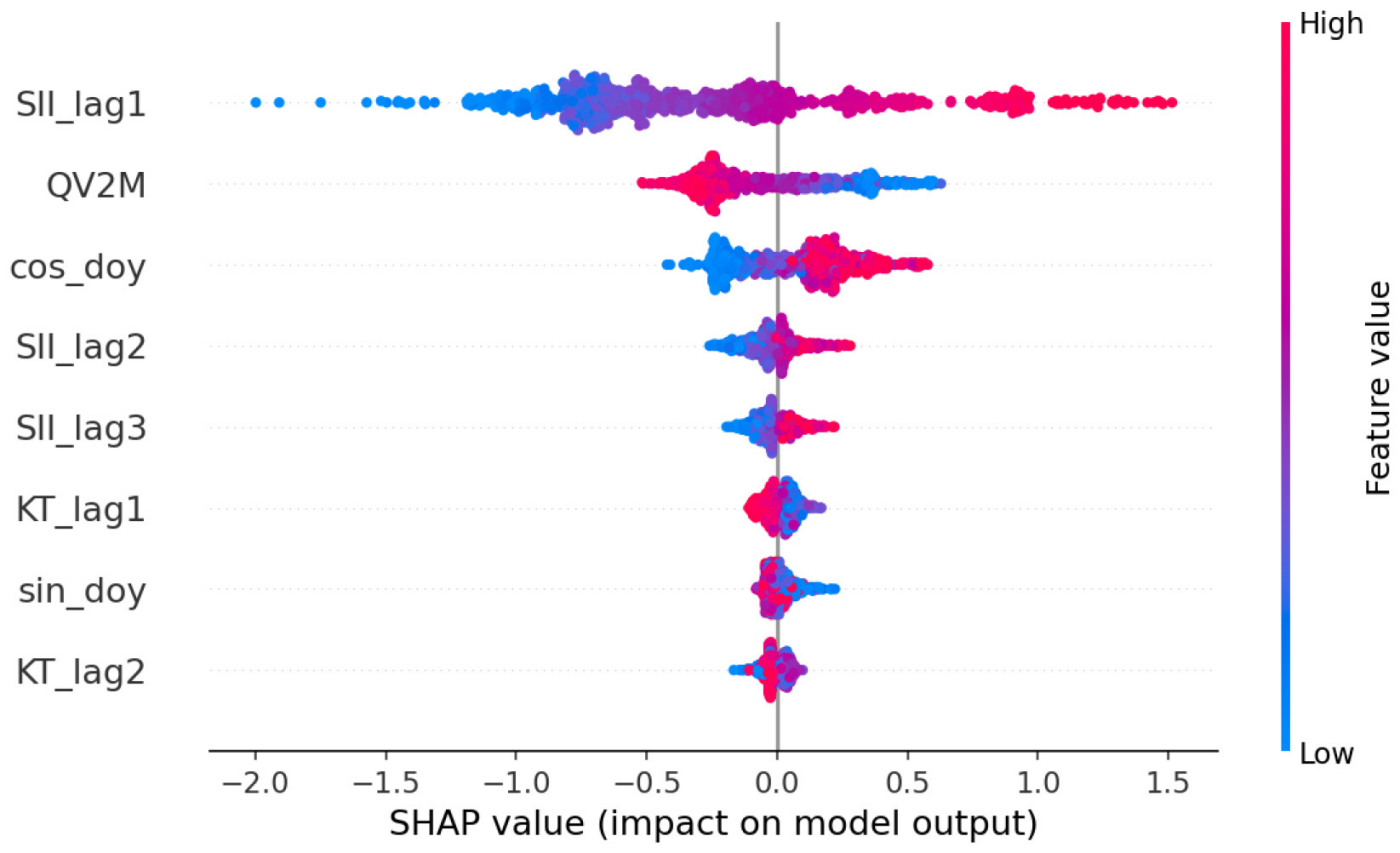
SHAP summary plot for the Random Forest model. Each point represents one test-day observation. Color indicates the feature value, with red representing high values and blue representing low values, while horizontal position represents the contribution to the predicted SII value.

Figure 7 compares the impurity-based importance of the Random Forest with the permutation importance calculated for the held-out test set. Both approaches identified the previous-day irradiation (SII_lag1_) and previous-day specific humidity (QV2M_lag1_) as the most influential predictors. In contrast, the lagged clearness index variables made comparatively smaller contributions. The agreement between the two importance measures provides additional support for the dominant role of recent irradiation conditions and atmospheric humidity in Random Forest predictions.

The SHAP summary plot in Figure 8 complements the global importance results in Figure 7 by showing both the magnitude and direction of each predictor's contribution. Each point represents one observation from the test period, and its horizontal position indicates whether the predictor increased or decreased the corresponding SII prediction accuracy. The distribution of SHAP values confirmed that SII_lag1_ and QV2M exerted the largest effects on individual predictions, whereas the remaining lagged and seasonal variables had more limited contributions.

The predictive behaviors of the two leading representative models were further examined using the observed-vs.-predicted and residual diagnostic plots shown in Figure 9. The observed-vs.-predicted panels illustrate the extent to which Linear Regression and Random Forest reproduce the variability of the measured irradiation values. The residual-vs.-predicted panels provide evidence of systematic underprediction, overprediction, and possible changes in error variance across the prediction range.

**Figure 9:**
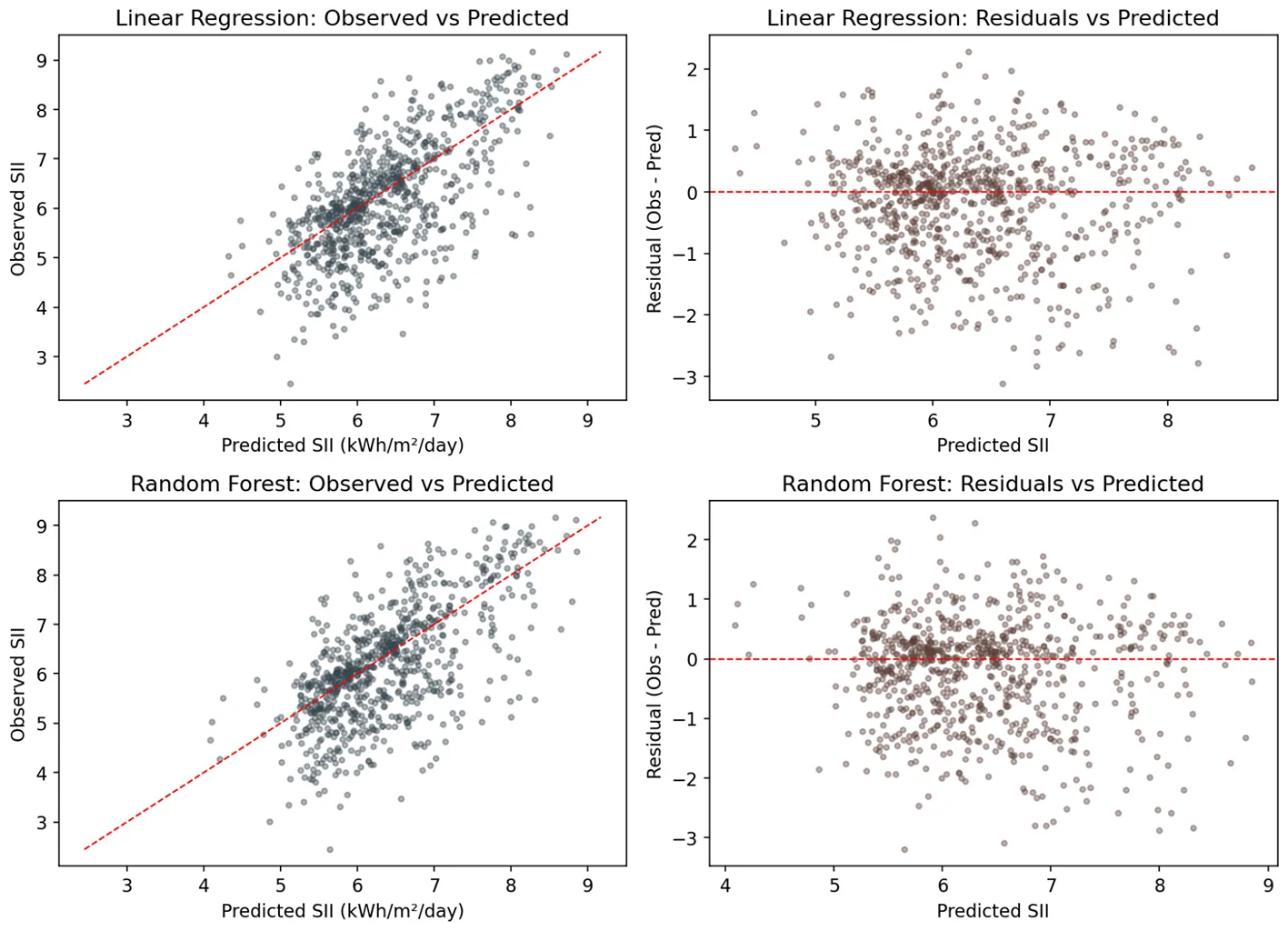
Observed-vs.-predicted and residual-vs.-predicted plots. Diagnostic results are shown for Linear Regression and Random Forest on the held-out test set.

As shown in Figure 9, both models reproduced the central range of the observations more accurately than the most extreme irradiation values. The residual dispersion indicates that part of the day-to-day variability remained unexplained by the available lagged, humidity, and seasonal predictors. This diagnostic behavior is consistent with the moderate *R*^2^ values presented in Table 5.

Figure 10 compares the distributions of the absolute prediction errors for all six models. The substantial overlap among the boxes and whiskers confirms that the algorithms produced similar error distributions, which is consistent with the small differences in the MAE and RMSE values reported in Table 5. Linear Regression presented a marginally more favorable central error distribution; however, no model demonstrated a clearly separated advantage over the others.

**Figure 10:**
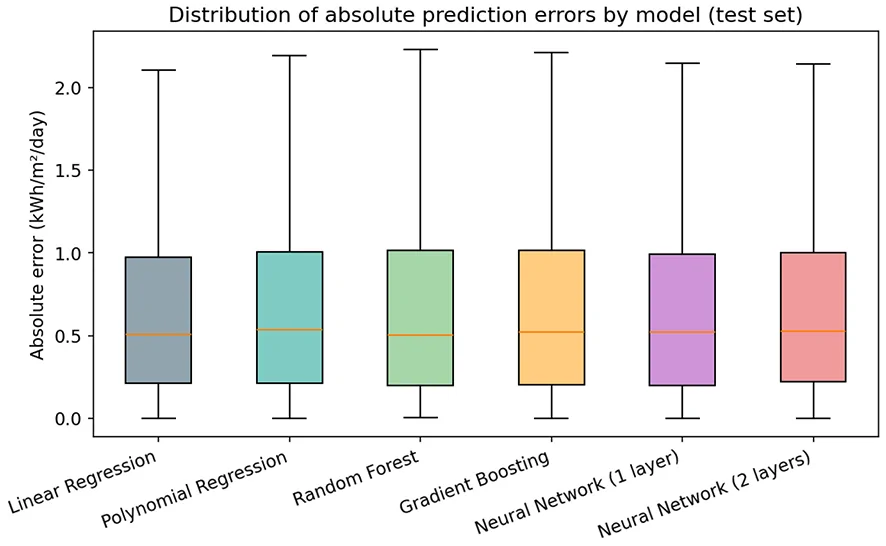
Distribution of absolute test-set errors. Absolute prediction errors are compared across the six models. Values beyond the whiskers are omitted from the visualization to improve readability.

The Random Forest learning curve presented in Figure 11 was used to assess whether the model performance was constrained primarily by the available sample size. The cross-validated MAE decreased as the training sample initially increased but began to stabilize after approximately 300 daily observations. The persistence of a gap between the training and validation errors, together with the subsequent validation plateau, indicates that adding more observations from the same predictor structure would probably produce only limited improvements in the model performance.

**Figure 11:**
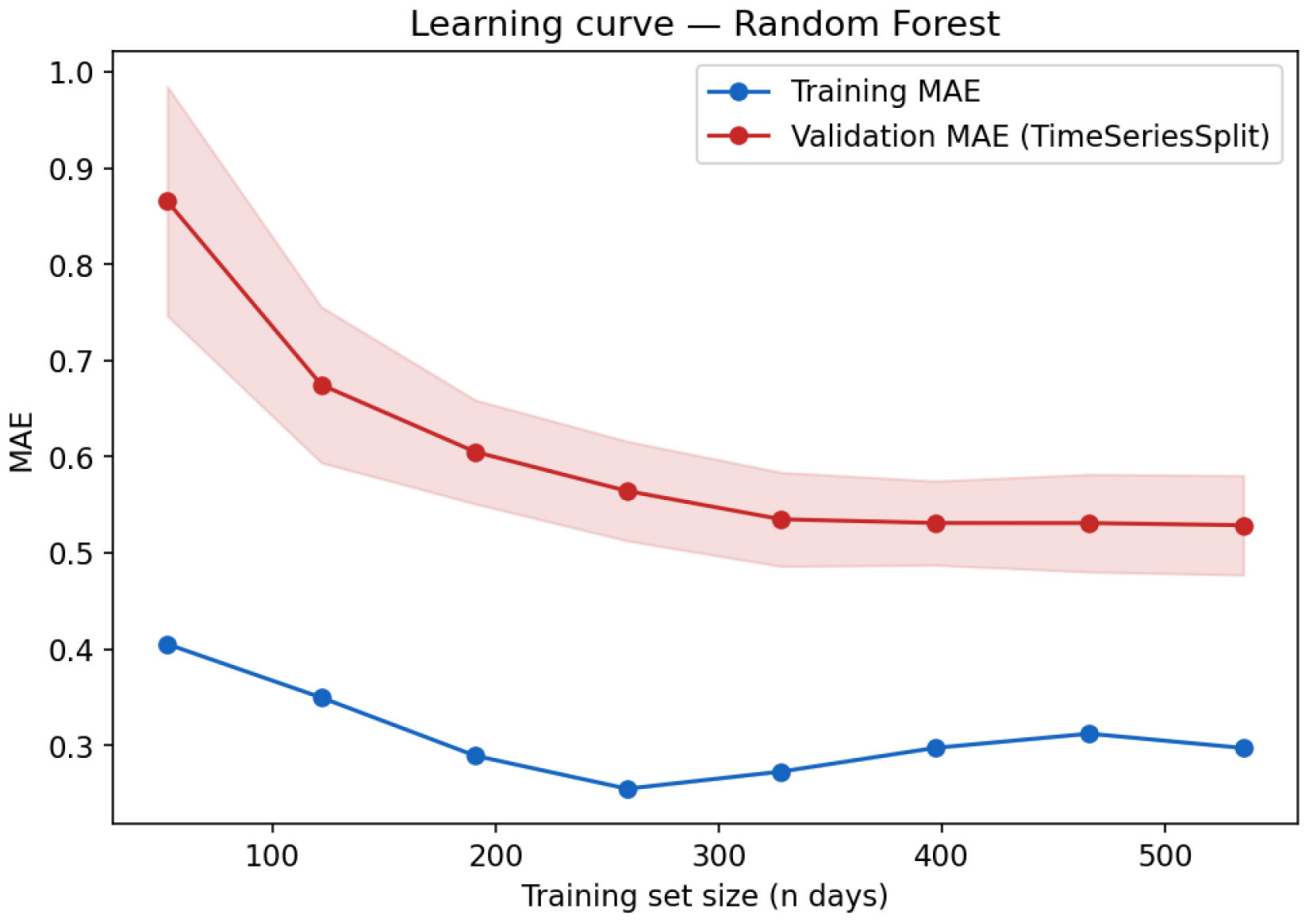
Random Forest learning curve. Training and cross-validated MAE obtained using five-fold TimeSeriesSplit are presented as functions of training-set size. The validation error stabilizes after approximately 300 daily observations.

Therefore, Figure 11 suggests that the principal limitation is not simply an insufficient number of training observations. Instead, further performance gains may require additional informative meteorological predictors, higher temporal resolution, improved measurements, and variables that can represent rapidly changing atmospheric conditions.

The Taylor diagram in Figure 12 provides a complementary assessment by simultaneously comparing the correlations between the models and the observations and their standard deviations. All six models occupy a relatively compact area in the diagram, demonstrating their similar predictive behavior. Linear Regression was positioned closest to the observational reference according to the combined correlation and variability criteria, although the differences from the ensemble and neural-network models remained limited.

**Figure 12:**
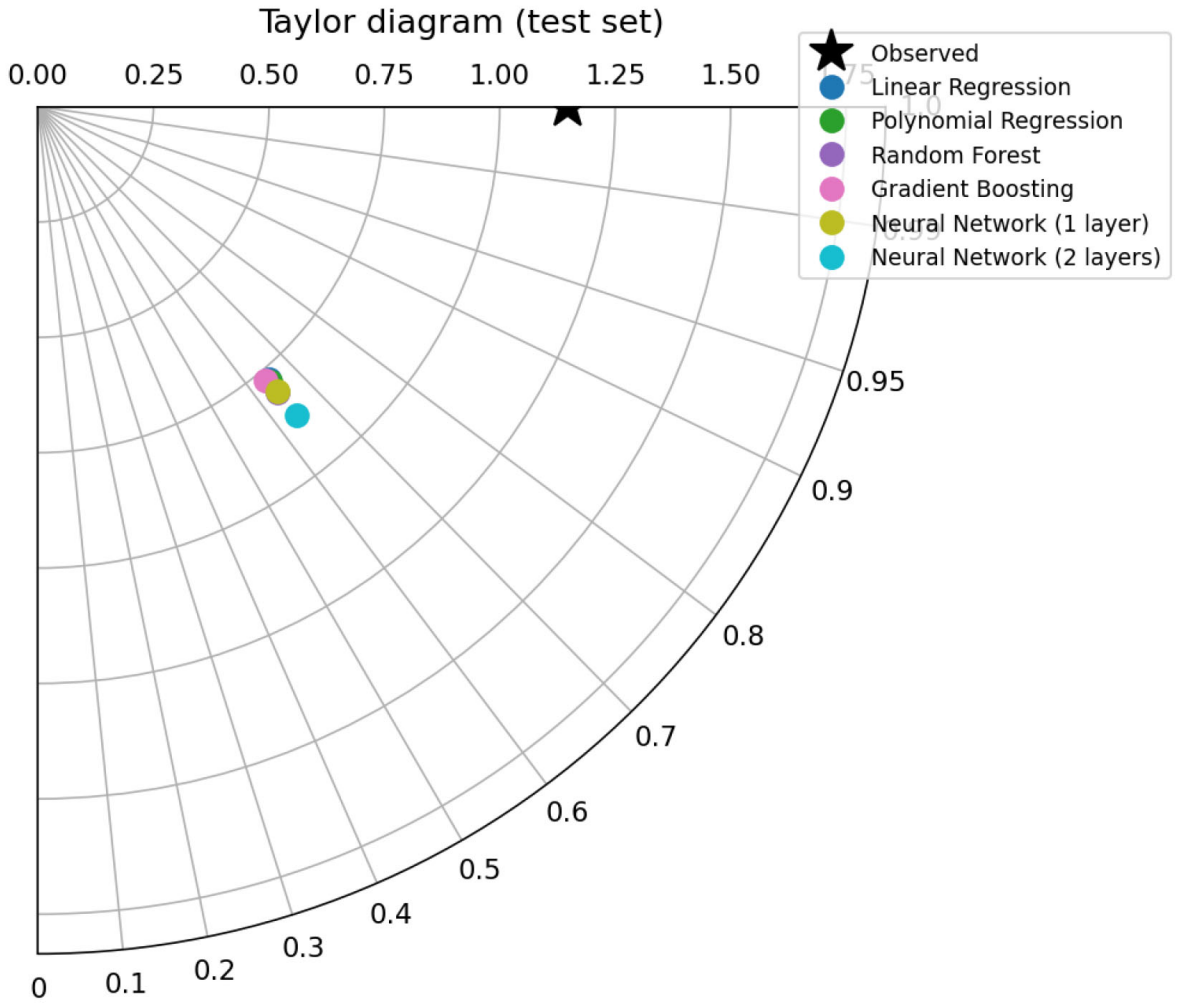
Taylor diagram for the six forecasting models. The diagram summarizes the standard deviation and correlation of each model relative to the observed test-set values, represented by the black star.

Figure 13 integrates the five evaluation metrics into a normalized multi-criteria comparison. For visualization purposes, the MAE, RMSE, MAPE, and SMAPE were inverted so that larger normalized values consistently represented better performance, as in the case of *R*^2^. The resulting profiles show that Linear Regression provides the most balanced performance across the complete set of metrics. Gradient Boosting and Random Forest remain close competitors, whereas the neural network with two hidden layers presents the least favorable overall profile.

**Figure 13:**
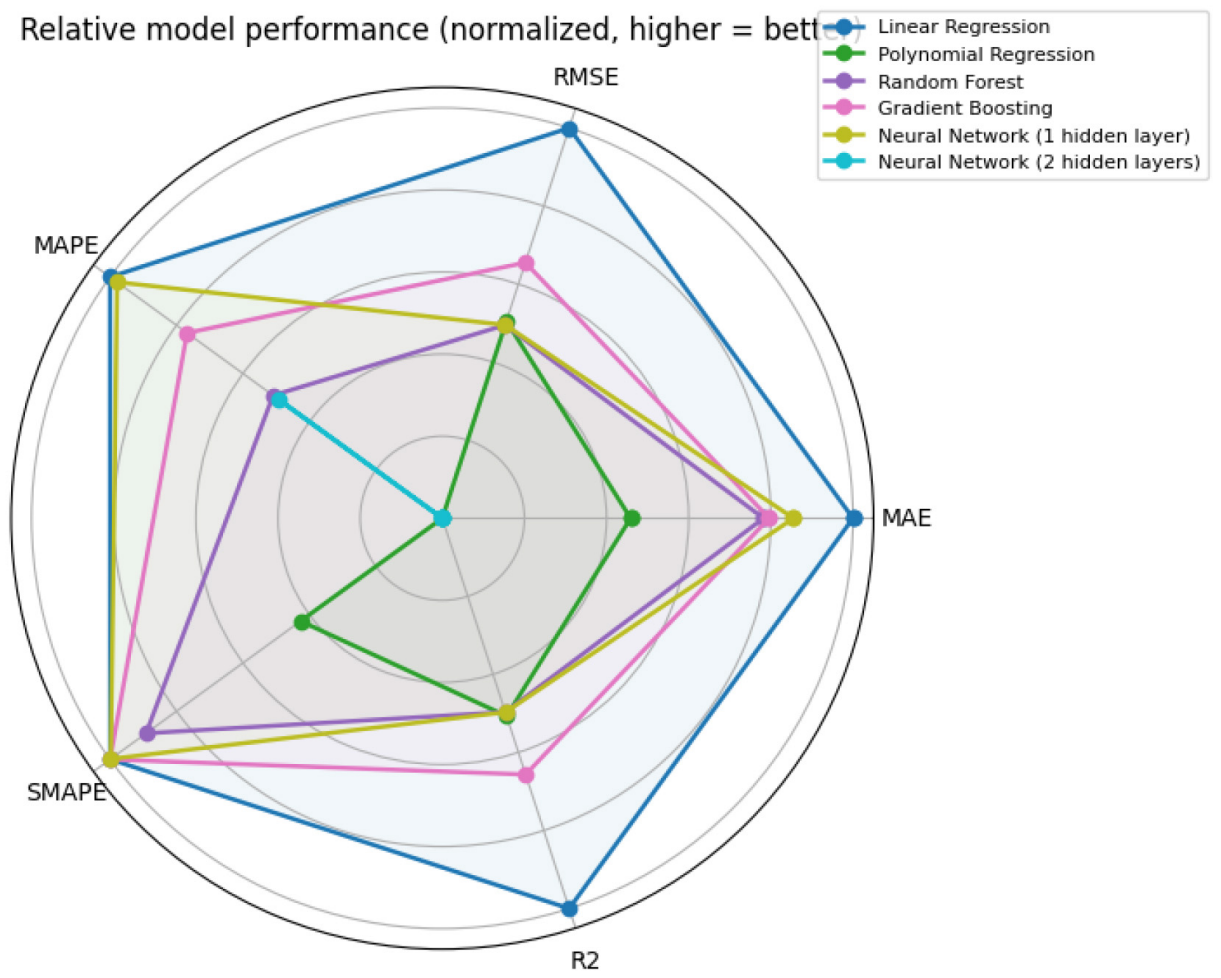
Normalized multi-metric model comparison. MAE, RMSE, MAPE, and SMAPE are inverted so that larger values indicate better performance, consistent with the interpretation of *R*^2^. Linear Regression presents the most balanced overall profile.

The agreement in Table 5, the direct metric visualization in Figure 6, the Taylor diagram in Figure 12, and the radar chart in Figure 13 strengthen the conclusion that model complexity does not yield a meaningful predictive advantage in this application.

To place these findings in a broader empirical context, Table 6 compares the performance of this study with the results reported in previous solar-radiation forecasting studies. This comparison must be interpreted cautiously because the studies differ in terms of location, altitude, sample size, observation frequency, predictor availability, temporal coverage, validation strategy, and reported metrics.

**Table 6:** Comparison between the present results and previous solar-radiation forecasting studies cited in the Introduction and Discussion.

Study	Location/sample	Best model	*R* ^2^	RMSE	MAE
Present study	Juliaca, Peru, >3,800 m (*n* = 4, 000, daily observations)	Linear regression	0.390	0.896	0.677
Gala et al. (2016)	Rajasthan, India	Random Forest	Not reported	0.64 (19.5% better than Linear Regression)	*n/a*
Oviedo et al. (2023)	UTEQ campus, Ecuador	Gradient boosting	Reported as high; exact value not available	*n/a*	*n/a*
Demir (2025)	Konya, Türkiye, 1984–2022	LSTM/Bi-LSTM	0.97	0.34/0.37	0.27/0.31
Zhang et al. (2025)	China; hourly observations under fog–haze conditions	XGBoost + AQI	*R* reported instead of *R*^2^	0.28–0.32 (normalized)	*n/a*

Differences in temporal resolution, study period, predictors, validation design, and reporting metrics should be considered when interpreting the results.

As shown in Table 6, the *R*^2^ obtained in this present study is lower than the values reported in some previous investigations, particularly the LSTM and Bi-LSTM results reported by Demir (2025). However, those results were obtained using a substantially longer historical record covering 1984–2022, whereas the present analysis used daily observations from 2010 to 2020 and implemented strict controls against same-day target leakage. Moreover, the studies summarized in Table 6 were conducted under different climatic, geographic, and methodological conditions, which prevented direct comparisons.

Nevertheless, the contrast with previous studies identifies potential directions for future research to explore. In particular, the long-term dataset used by Demir (2025) provides a benchmark for evaluating the LSTM and Bi-LSTM architectures at the Juliaca station once a comparably extensive record is available. Similarly, the results reported by Zhang et al. (2025) illustrated the potential value of incorporating atmospheric-quality variables under adverse conditions, although their hourly and normalized metrics are not directly comparable with the daily metrics used in the present study.

Overall, the evidence from Table 5, Figures 6–13, and Table 6 consistently indicates that Linear Regression achieved the best numerical results, whereas Gradient Boosting and Random Forest remained closely competitive. The narrow differences among the leading models demonstrate that the predictive performance was not determined solely by algorithmic complexity. Instead, the quality, temporal validity, and physical relevance of the predictors appear to be more influential than the use of increasingly complex learning algorithms.

Linear Regression achieved the best numerical performance, although its advantage over Gradient Boosting and Random Forest was small. The *R*^2^ difference between Linear Regression and Gradient Boosting was 0.006, whereas their MAE values differed by only 0.001 kWh m^−2^d^−1^. Therefore, the results do not support the claim of decisive superiority for either neural network or ensemble-based approaches. The competitive performance of Polynomial Regression also indicates that the relationship between the legitimate predictors and next-day irradiation contains a structure that can be represented without relying exclusively on highly complex models. The random forest model remained competitive and offered greater interpretability through impurity-based importance, permutation importance, and SHAP analysis. Consequently, model selection should consider not only predictive accuracy but also interpretability, computational costs, and operational requirements. Variable selection is a critical determinant of predictive accuracy. Previous studies have identified additional variables, including surface temperature, wind speed, and atmospheric pressure, as influential predictors (Hissou et al., 2024; Mariappan et al., 2025). Incorporating these variables, combined with dimensionality reduction techniques such as principal component analysis, may enhance the predictive performance by prioritizing the most informative features.

The performance obtained in the present study is lower than the values reported in some previous solar-radiation forecasting studies, such as Gala et al. (2016) and Oviedo et al. (2023). However, direct comparisons should be interpreted cautiously because the studies differ in forecasting horizos, predictor availability, temporal resolution, geographic conditions, sample size, and potential exposure to target-derived predictors. Therefore, the principal contribution of the present analysis is not an exceptionally high *R*^2^, but a more credible evaluation obtained after identifying and excluding predictors that could inflate the apparent forecasting performance.

Accurate solar radiation prediction supports the efficient planning and operation of renewable energy systems by reducing the uncertainty in energy availability. The models developed in this study provide reliable estimates that facilitate integration into power grids and support informed decision-making, thereby improving energy management efficiency (Oviedo et al., 2023).

The Random Forest interpretation results in Figures 7, 8 show that previous-day irradiation and specific humidity provided most of the predictive information, whereas the lagged clearness index variables made smaller contributions. This pattern may be consistent with atmospheric processes that are characterized by limited persistence. A plausible physical explanation is that, above 3,800 m a.s.l., the reduced atmospheric column and lower aerosol loading limit the day-to-day variability introduced by scattering and absorption, so that the dominant remaining source of variation is intermittent convective cloud cover, whose effects tend to persist for one to a few days. This would explain why lag-1 irradiance and lag-1 humidity, which are closely tied to local convective activity in the Andean altiplano, outperform the lagged clearness-index terms as predictors. However, because the dataset does not include direct cloud observations, cloud type, or synoptic classifications, this mechanism remains a plausible interpretation rather than an established explanation and cannot be confirmed from the present analysis alone.

The relatively narrow range of test performance (*R*^2^ = 0.372–0.390) suggests that the legitimate predictor set contains limited additional information that can be exploited by increasing model complexity. Rather than demonstrating an intrinsic predictability ceiling, these findings indicate a practical limitation associated with the variables and forecasting designs evaluated in this study.

### 3.1 Limitations and future work

This study did not include deep sequence models, such as LSTM, Bi-LSTM, or CNN–LSTM. (Demir, 2025) reported *R*^2^ = 0.97 for LSTM and Bi-LSTM models applied to a comparable daily NASA POWER time series; however, that study used a 38-year record covering 1984–2022, which is approximately 3.5 times longer than the 11-year record available here. Deep recurrent architectures generally require large datasets and, given the current sample size, would present a substantial risk of overfitting relative to the regression, ensemble, and shallow neural-network models evaluated in this study. Therefore, acquiring a longer historical record and subsequently benchmarking the LSTM and Bi-LSTM models represents the highest-priority extension of this research.

The predictor set also remained limited to lagged specific humidity, lagged irradiance, lagged clearness-index values, and solar-geometry variables. Additional meteorological variables, including surface temperature, wind speed, atmospheric pressure, and cloud fraction, were not available in the current NASA POWER extract for the Juliaca station and should be considered in future data acquisition and model development efforts.

Future research should address these limitations by systematically expanding the variables, models, and data sources. Incorporating additional meteorological predictors, including surface temperature, wind speed, atmospheric pressure, cloud cover, and precipitation, would improve the representation of atmospheric processes and enhance the predictive robustness. The inclusion of deep learning architectures, such as LSTM and Bi-LSTM, would enable more comprehensive comparisons, particularly in capturing the temporal dependencies in solar radiation time series. Expanding the spatial scope to multiple regions would facilitate the evaluation of model generalizability under heterogeneous climatic conditions and support the identification of spatial patterns in predictive performance. Moreover, integrating higher-resolution data from local weather stations, remote sensing platforms, and sensor networks would improve the detection of microclimatic variability and short-term fluctuations, thereby increasing the model accuracy. The incorporation of climate change scenarios and related variables would further strengthen long-term forecasting by accounting for non-stationary conditions. In parallel, embedding predictive models into real-time energy management systems would enhance operational decision-making by enabling dynamic adjustments to the energy supply and demand. Collectively, these research directions will contribute to the development of more accurate, scalable, and adaptive solar radiation forecasting models for renewable energy systems.

## 4 Conclusion

This study compared six regression and machine learning models for 1-day-ahead prediction of daily global horizontal irradiation at a high-altitude Andean site in Juliaca, Peru. Under the leakage-controlled forecasting design, Linear Regression achieved the best numerical test performance, with an MAE of 0.677 kWh m^−2^d^−1^, an RMSE of 0.896 kWh m^−2^d^−1^, and an *R*^2^ of 0.390. Gradient Boosting and Random Forest produced closely comparable results, with *R*^2^ values of 0.384 and 0.381, respectively. The small performance differences among the leading models indicate that the results do not demonstrate the decisive superiority of any particular model. Instead, they showed that simple linear regression, Gradient Boosting, and Random Forest can provide comparable forecasts when trained with the same temporally ordered predictor set. Therefore, model selection should consider interpretability and computational requirements in addition to predictive accuracy. Incorporating additional astronomical variables that describe the Sun-Earth geometry, including the declination angle, hour angle, zenith angle, elevation angle, and azimuth angle, may further improve the physical basis of future models.

The main methodological contribution is the identification of the leakage risk introduced by same-day clearness-index predictors, which are algebraically related to the target irradiation value. Excluding this information produced more modest but more credible out-of-sample estimates than the previous leakage-affected model. Future studies should evaluate additional forecast-available meteorological variables, ground-based measurements, longer temporal records, and multiple high-altitude sites before making broader claims regarding operational or geographical generalizability of the findings.

## Data Availability

Publicly available datasets were analyzed in this study. This data can be found here: https://acesse.one/8fv2rhy.

## References

[B1] Alsharif M. H. Younes M. K. Kim J. (2019). Time series ARIMA model for prediction of daily and monthly average global solar radiation: the case study of Seoul, South Korea. Symmetry 11:240.

[B2] AutoSolar (2025). Peru: Highest Solar Radiation on the Planet. Available online at: https://autosolar.pe/energia-solar/peru-radiacion-solar-mas-alta-de-todo-el-planeta (Accessed April 15, 2026).

[B3] Bamisile O. Cai D. Oluwasanmi A. Ejiyi C. Ukwuoma C. C. Ojo O. . (2022). Comprehensive assessment, review, and comparison of AI models for solar irradiance prediction based on different time/estimation intervals. Sci. Rep. 12:9644. 35688900 10.1038/s41598-022-13652-w PMC9187635

[B4] Belmahdi B. Louzazni M. Marzband M. El Bouardi A. (2023). Global solar radiation forecasting based on hybrid models with combinations of meteorological parameters. Forecasting 5, 172–195. doi: 10.3390/forecast5010009

[B5] Breiman L. (2001). Random forests. Mach. Learn. 45, 5–32. doi: 10.1023/A:1010933404324

[B6] Chodakowska E. Nazarko J. Nazarko Ł. (2023). Arima models in solar radiation forecasting: a systematic review. Energies 16:1922. doi: 10.3390/en16135029

[B7] Demir V. (2025). Evaluation of solar radiation prediction models using AI: a performance comparison in the high-potential region of Konya, Türkiye. Atmosphere 16:398. doi: 10.3390/atmos16040398

[B8] Friedman J. H. (2001). Greedy function approximation: a gradient boosting machine. Ann. Stat. 29, 1189–1232. doi: 10.1214/aos/1013203451

[B9] Gala Y. Fernández Á. Díaz J. Dorronsoro J. R. (2016). Hybrid machine learning forecasting of solar radiation values. Neurocomputing 176, 48–59.

[B10] Gupta R. Yadav A. K. Jha S. K. (2024). Harnessing the power of hybrid deep learning algorithm for the estimation of global horizontal irradiance. Sci. Tot. Environ. 943:173958. doi: 10.1016/j.scitotenv.2024.173958 38871320

[B11] He K. Zhang X. Ren S. Sun J. (2016). “Deep residual learning for image recognition,” in Proceedings of the IEEE conference on computer vision and pattern recognition (CVPR) (Las Vegas, NV: IEEE), 770–778. doi: 10.1109/CVPR.2016.90

[B12] Hinton G. E. Osindero S. Teh Y.-W. (2006). A fast learning algorithm for deep belief nets. Neural Comput. 18, 1527–1554. doi: 10.1162/neco.2006.18.7.1527 16764513

[B13] Hissou H. Benkirane S. Guezzaz A. Azrour M. Beni-Hssane A. (2024). A lightweight time series method for prediction of solar radiation. Energy Syst. 17, 127–164. doi: 10.1007/s12667-024-00657-9

[B14] Infield D. Freris L. (2020). Renewable Energy in Power Systems, 2nd edn. Chichester: John Wiley & Sons.

[B15] International Energy Agency (2020). World Energy Outlook 2020.

[B16] Mariappan Y. Ramasamy K. Velusamy D. (2025). An optimized deep learning based hybrid model for prediction of daily average global solar irradiance using CNN-SLSTM architecture. Sci. Rep. 15:10761. doi: 10.1038/s41598-025-95118-3 40155655 PMC11953468

[B17] Menacho-Chiok C. H. (2014). Estadística aplicada a la investigación científica. Lima: Universidad Nacional Mayor de San Marcos.

[B18] Nadeem T. B. Ali S. U. Asif M. Suberi H. K. (2024). Forecasting daily solar radiation: an evaluation and comparison of machine learning algorithms. AIP Adv. 14:075010. doi: 10.1063/5.0211723

[B19] NASA (2025). Nasa Power Data Access Viewer. Available online at: https://power.larc.nasa.gov/ (Accessed April 15, 2026).

[B20] Nastic F. Jurisevic N. Nikolic D. Koncalovic D. (2024). Harnessing open data for hourly power generation forecasting in newly commissioned photovoltaic power plants. Energy Sustain. Dev. 81:101512. doi: 10.1016/j.esd.2024.101512

[B21] Oviedo B. Anchundia Troncoso J. Torres Quijije A. Zambrano-Vega C. (2023). Solar radiation prediction in the UTEQ based on machine learning models. Lect. Notes Netw. Syst. 1055, 79–89. doi: 10.1007/978-981-97-5441-0_8

[B22] Pachauri R. K. Meyer L. A. (2014). Climate Change 2014: Synthesis Report. Contribution of Working Groups I, II and III to the Fifth Assessment Report of the Intergovernmental Panel on Climate Change. Geneva: IPCC.

[B23] Pang Z. Niu F. O'Neill Z. (2020). Solar radiation prediction using recurrent neural network and artificial neural network: a case study with comparisons. Renew. Energy 156, 279–289.

[B24] Panjwani M. K. Narejo G. B. (2014). Effect of humidity on the efficiency of solar cell (photovoltaic). Int. J. Eng. Res. Gen. Sci. 2, 499–503.

[B25] Photovoltaic Software (2025). How to Calculate the Annual Solar Energy Output of a Photovoltaic System. Available online at: https://photovoltaic-software.com/principle-ressources/how-calculate-solar-energy-power-pv-systems (Accessed April 15, 2026).

[B26] Quispe Huamán L. Sotomayor Alzamora G. (2022). Determinación y análisis temporal de la radiación solar global en el Altiplano de Puno. Ingeniare. Revista chilena de ingeniería 30, 69–81.

[B27] Rumelhart D. E. Hinton G. E. Williams R. J. (1986). Learning representations by back-propagating errors. Nature 323, 533–536. doi: 10.1038/323533a0

[B28] Tampubolon D. K. F. Khayam U. Isnandar S. Banjar-Nahor K. M. Inkaresa A. Laksono F. A. . (2026). Project risk assessment of renewable energy projects in electricity market structures: a systematic literature review. Energies 19:3179.

[B29] Teke A. Yildirim H. B. Celik O. (2015). Evaluation and performance comparison of different models for the estimation of solar radiation. Renew. Sustain. Energy Rev. 50, 1097–1107. doi: 10.1016/j.rser.2015.05.049

[B30] Tsai W. C. Tu C. S. Hong C. M. Lin W. M. (2023). A review of state-of-the-art and short-term forecasting models for solar PV power generation. Energies 16:5436.

[B31] Wald L. (2018). Fundamentals of Solar Radiation. Boca Raton, FL: CRC Press.

[B32] Wei C. C. (2017). Predictions of surface solar radiation on tilted solar panels using machine learning models: a case study of Tainan City, Taiwan. Energies 10:1660. doi: 10.3390/en10101660

[B33] Weyll A. L. C. Kitagawa Y. K. L. Araujo M. L. S. Ramos D. N. S. Lima F. J. L. Santos T. S. . (2024). Medium-term forecasting of global horizontal solar radiation in Brazil using machine learning-based methods. Energy 300:131549. doi: 10.1016/j.energy.2024.131549

[B34] Yang X. Ji Y. Wang X. Niu M. Long S. Xie J. (2023). Simplified method for predicting hourly global solar radiation using extraterrestrial radiation and limited weather forecast parameters. Energies 16:3215. doi: 10.3390/en16073215

[B35] Zhang C. Zhang Y. Pu J. Liu Z. Wang Z. Wang L. (2025). Hourly solar radiation prediction using extreme gradient boosting with fog-haze effects. Energy Built Environ. 6, 18–26. doi: 10.1016/j.enbenv.2023.08.001

